# Comparing Gender Differences in Willingness to Accept Same- and Other-Sex Dyadic and Multi-Person Sexual Offers: An Examination of the Backlash Effect

**DOI:** 10.3390/bs15081128

**Published:** 2025-08-20

**Authors:** Ashley E. Thompson, Lizzy Bensen, Ryan Scoats

**Affiliations:** 1 Department of Psychology, University of Minnesota Duluth, Duluth, MN 55812, USA; ward1089@d.umn.edu; 2 Department of Sociology, Birmingham City University, Birmingham B5 5JU, UK; ryanscoatsphd@gmail.com

**Keywords:** sexual double standard, backlash effect, mixed-sex threesomes

## Abstract

Proponents of Sexual Script Theory posit that men and women differ in their sexual behaviors/motivations, often due to culturally ingrained expectations. When these expectations are violated, individuals may face stigma, particularly in nontraditional contexts like mixed-sex threesomes (MSTs). This study examined gender differences in heterosexual adults’ anticipated stigma and willingness to accept various sexual offers, including dyadic and MST offers involving same- and other-sex partners, through the lens of the backlash effect (i.e., the anticipation of stigma for participating in sexual behaviors that contradict societal expectations). A total of 540 heterosexual U.S. adults read vignettes depicting hypothetical sexual invitations and completed measures assessing anticipated stigma and willingness to engage. Results revealed that men anticipated less stigma and were more willing to accept sexual offers than women. Participants also anticipated less stigma and reported greater willingness to accept other-sex dyadic offers as compared to same-sex or MST offers. However, men reported the highest anticipated stigma and lowest willingness for same-sex dyadic offers, suggesting a novel backlash effect against men engaging in gender non-conforming behaviors. These findings offer support for the persistence of the sexual double standard and provide evidence for gendered backlash effects, including those impacting heterosexual men.

## 1. Introduction

Stigma often occurs in response to the violation of scripts, particularly sexual scripts (i.e., the shared beliefs and expectations held amongst a particular social group that guides the sexual behaviors of the individuals within that group; Simon & Gagnon, 1984). Proponents of Sexual Script Theory argue that men tend to value shorter-term relationships and adopt a pleasure-oriented approach when pursuing relationships, whereas women often value longer-term relationships and adopt an emotionally oriented approaches to relationships (Bowleg et al., 2004; Frith & Kitzinger, 2001). When these gendered socially constructed expectations are not upheld, double standards can form and cause men and women to be evaluated differently, resulting in the traditional sexual double standard (SDS; defined as the tendency for women to be judged more harshly than men for comparable sexual behavior; Papp et al., 2015).

Despite the robust literature documenting the presence of the traditional SDS (wherein women are sanctioned for sexual agency and men are rewarded; Papp et al., 2015), recent research has suggested a more complex reality. In fact, some studies suggest that the SDS has weakened or become more egalitarian over time (e.g., Milhausen & Herold, 2002; Marks & Fraley, 2005), whereas others have identified evidence of an SDS favoring men over women (Hensums et al., 2020; Thompson et al., 2018).

Multiple explanations have been proposed to account for these inconsistencies in the literature. Chief among them are (1) measurement concerns related to the over-reliance on explicit self-report measures, which are often plagued by social desirability biases and (2) the possibility that the SDS is situationally specific and less pronounced in some domains. For example, research exploring the endorsement of an implicit SDS and situationally specific judgments continues to uphold traditional gendered sexual norms (e.g., England & Bearak, 2014; Kreager et al., 2016; Thompson et al., 2020). In one such study, Thompson et al. (2020) found that participants demonstrated an implicit preference for sexual stimuli when primed with male versus female targets. These findings suggest that sexual behavior is more automatically associated with men, a bias reflective of the SDS, and highlight the value of implicit methods, which may uncover patterns of stigma that are obscured or underreported in explicit measures.

### 1.1. Threesome Participation and the SDS

Additionally, there is some evidence that the SDS is most prominent when evaluating those participating in nontraditional sexual behaviors, such as mixed-sex threesomes (MSTs) (i.e., sexual activity involving three people at the same time, during which persons of more than one sex are present; Thompson et al., 2022). In fact, research indicates that hypothetical women engaging in MSTs are evaluated more negatively than are men (Jonason & Marks, 2009; Thompson & Byers, 2021). For example, in a study by Jonason and Marks (2009), university students were presented with hypothetical scenarios that described either a man or a woman engaging in an MST. After reading the scenario, participants were asked to rate the extent to which a variety of derogatory labels (e.g., dirty, selfish, whore) accurately described the target man or woman. It was found that the hypothetical woman target was assigned derogatory labels to a significantly greater extent than the man target for engaging in an MST.

Additionally, Thompson and Byers (2021) presented U.S. adults with hypothetical scenarios in which male or female targets initiated an MST and then asked participants to rate the targets on a variety of character constructs. Findings revealed that hypothetical male initiators were rated more favorably in their cognitive abilities, morality, and quality as a partner than hypothetical female initiators. Furthermore, when the targets initiated an MST in a casual sexual context (rather than in the context of a committed relationship), female initiators were judged as having more extensive sexual histories than male initiators. Thus, the authors present a case for the continued existence of the traditional SDS in regard to MSTs.

### 1.2. Sex, Gender, and the Backlash Effect

Although the two studies exploring the SDS with regard to MSTs expanded our understanding on this topic, the impact of MST participation in light of the SDS remains unclear. In fact, it is possible that MST participants—particularly women—may develop a fear of being stigmatized, a phenomenon which has been defined as the *backlash effect* (Conley et al., 2013; Rudman, 1998). Although the backlash effect has rarely been applied in sexuality-related research, it has regularly been used to explain and understand gender differences in workplace settings. In fact, social backlash against women is common in the workplace, as power-seeking women often face more negative social consequences than do power-seeking men (e.g., Brescoll et al., 2018; Mishra & Kray, 2022). Thus, research has revealed that women may avoid engaging in workplace behaviors that are likely to result in stigma (e.g., assertive salary negotiations, describing oneself as competitive in job applications; Amanatullah & Morris, 2010; Moss-Racusin & Rudman, 2010; Williams & Tiedens, 2016).

The backlash effect is also not limited to women, as men too are subject to social penalties when violating gender expectations. Moss-Racusin et al. (2010) found that men who demonstrated modesty rather than socio-culturally expected agentic behaviors (i.e., dominance, self-promotion, etc.) in hypothetical job interview scenarios faced backlash in the form of being liked less than identically modest women. Men were perceived as violating status-related gender norms, showing insufficient confidence and excessive weakness. Similarly, when entering female-coded professions, such as elementary school teaching, Moss-Racusin and Johnson (2016) found that male applicants were perceived as more likely to be gay and to pose a greater threat to children, and were again rated as less likeable than identical female applicants. Some research has pointed to political conservatism and its accompanying motivation to maintain the status quo—rather than solely to endorsements of more traditional beliefs around masculinity—which contribute toward backlash against men displaying female-coded behaviors and characteristics (Iacoviello et al., 2021).

Looking to the research on violations of sexual expectations, Conley et al. (2013) explored attitudes toward those who accepted offers of casual sex, both toward hypothetical others and how the participants themselves believed they would be perceived. Across four different studies and close to 3000 participants, they discovered that (compared to men) women received and anticipated harsher judgments for accepting a casual sexual offer and were thus less likely to accept offers out of fear of backlash. Similarly, Klein et al.’s (2019) work exploring sexual script deviation found a backlash toward women’s sexual assertiveness. However, this research found sexually assertive behavior overall was evaluated negatively, and sexually timid individuals were viewed more favorably, regardless of gender. These findings point toward a broader conservative attitude toward sexual assertiveness rather than simply backlash for gender-non-conforming sexual scripts.

### 1.3. Stigma and Backlash in Same-Sex Sexual Behaviors

Beyond heterosexual sexual interactions, same-sex sexual behaviors may also result in a backlash effect, particularly if they occur within heteronormative social arenas which promote traditional gender roles and sexual scripts. This is especially true for men (Mize & Manago, 2018). Within contemporary Western societies, traditional gender roles for men have historically carried an expectation of heterosexuality and men’s same-sex sexual interactions have thus been discouraged through the application of stigma and homophobia (Anderson, 2011). Thus, men have tended to distance themselves from same-sex sexual acts (or hide their engagement in such acts) for fear of the stigma and loss of status they would be subject to (Anderson, 2005; Kimmel, 1994; Pascoe, 2007). That said, there is research to suggest that, for some heterosexual men, the boundaries of these sexual and gendered scripts are no longer strictly adhered to in the ways they once were (Carrillo & Hoffman, 2018; Reback & Larkins, 2010; Scoats et al., 2018). However, these changes are far from universal, and men who adhere to more conservative gendered scripts (in Western countries at least) will still eschew and stigmatize same-sex sexual contact and identities (Bettinsoli et al., 2020; McCormack & Anderson, 2014).

In contrast, women’s same same-sex sexual interactions have been much less rigorously policed or stigmatized (Worthen, 2013), and attitudes toward lesbian women have tended to be more positive than attitudes toward gay men (Herek, 2002). There are many potential factors which may influence this enhanced level of acceptance, for example, the (male) sexualization and objectification of women’s same-sex sexual behaviors (see Worthen, 2013). Alternatively, there may be greater cultural recognition and expectancy around women’s capacity for sexual fluidity/erotic plasticity (Diamond, 2008; Baumeister, 2000). As Brienne Fahs (2014) has argued, women’s performative bisexuality may be expected in some public contexts, and it does not necessarily challenge women’s heterosexual identities. Of course, this is not to say that women’s same sex-sexual behavior is always deemed acceptable; it just tends to be more accepted than men’s (Mize & Manago, 2018).

Although research on the SDS and backlash effects has primarily focused on heterosexual contexts, these phenomena may operate differently in some non-heterosexual interactions. Indeed, although research suggests that gender non-conforming gay men and lesbian women are subject to greater levels of backlash (see Worthen, 2013), many studies still highlight the malleability and diversity of sexual scripts and displays of gender across different non-heterosexual groups (Dolezal et al., 2024; Hoppe, 2011; Patterson et al., 2013; Scoats et al., 2021; Wignall, 2022). However, although sexual minorities might often have greater agency to reformulate sexual scripts that diverge from established heteronormative, gendered scripts, they may well still be influenced by them. For example, Matsick et al. (2021) found that gender differences in casual sex acceptance persisted between lesbian women and gay men, with gay men accepting offers more frequently. This difference was mediated by lesbian women’s heightened awareness of stigma surrounding casual sex within their own communities (rather than wider society more generally), suggesting that the SDS extends beyond heterosexual relationships and influences sexual decision-making even in same-sex contexts.

### 1.4. The Current Study

Thus, based on the literature documenting the many manifestations of the backlash effect, it is possible that it may also explain variations in men’s and women’s willingness to engage in MSTs with same- and other-sex partners. Thus, the current study was designed to examine gender differences in willingness to accept a variety of sexual offers (e.g., dyadic offers and MST offers) with both same- and other-sex partners. Using Sexual Script Theory (Simon & Gagnon, 1984) as a theoretical framework, the following hypotheses were generated:

**H1.**  *As compared to female participants, males were expected to report less anticipated stigma and greater willingness to accept sexual offers.*

**H2.**  *Participants were expected to report less anticipated stigma and greater willingness to accept dyadic sexual offers as compared to MST sexual offers.*

**H3.**  *Participants were expected to report less anticipated stigma and greater willingness to accept sexual offers that only included other-sex participants as compared to those that included a member of the same sex.*

**H4.**  *Anticipated stigma was expected to mediate the relationships between sex of participant, type of behavior, and sexual makeup and willingness to participate.*

**RQ:** Interaction effects were explored to determine if the impact of same-sex participation was greater for males as compared to females and if the impact of multiple partners was greater for females as compared to males.

## 2. Method

### 2.1. Participants

A total of 700 participants were recruited from two sources: 3Fun^®^ (a multi-person hook-up app; *N* = 259) and Prolific^®^ (an online crowdsourcing website; *N* = 281). All participants were required to be fluent English speakers, residents of the United States, at least 18 years of age, currently single, and identify as cisgender and heterosexual. After initial data cleaning, a total of 140 adults were removed due to incomplete participation, not meeting eligibility criteria, completing the survey too quickly (under two minutes), responding incorrectly to one of two attention check items, or for providing nonsensical responses to at least one of three open-ended items. Thus, the final sample comprised 540 U.S. adults (342 males, 198 females), with an average age of 34.12 (*SD* = 10.80). The majority of participants identified as White (46.1%), African American (23.0%), or Hispanic (19.1%). Just over half (51.6%) had previous experience with threesomes, and the average number of lifetime sexual partners was 29.33 (*SD* = 50.90).

### 2.2. Measures and Materials

#### 2.2.1. Experimental Vignettes

Eight vignettes were developed for the purposes of the current study. These vignettes manipulated two variables—the type of behavior (dyadic vs. threesome sex) and the sexual makeup of the behavior (all members of the other sex vs. one member of the same sex). In particular, the vignettes resembled a real-life scenario in which a hypothetical friend invited the respondent to participate in one of the sexual scenarios described above. See the Supplementary Materials for the full text included in all vignettes.

#### 2.2.2. Sexual Willingness Scale (SWS)

The SWS was developed for the purposes of the current study by modifying existing scales and developing items via focus groups with undergraduate students. In particular, four members of the principal investigator’s research team helped generate a pool of items by reviewing sample scales (Boot et al., 2016; Helmers et al., 2018) and posting on subreddits. The resulting scale comprised 12 items that were used in a pilot study with a convenience sample of 12 undergraduate students. Pilot participants were instructed to review each item for readability, grammatical accuracy, and clarity of meaning. In addition to providing feedback on wording, they were invited to suggest new items for inclusion, identify items that should be removed due to irrelevance or ambiguity, and recommend combining items they perceived as redundant or overly similar in content. This process ensured that the measure was both comprehensible to the target population and inclusive of relevant content domains.

Based on piloting, the final scale included eight items related to one’s excitement and motivation to accept the hypothetical sexual offer (*α* = 0.96). Sample items include “I would feel flattered by accepting the sexual offer” and “I would cancel plans (e.g., friends, family, skip work) to accept the sexual offer.” Instructions asked that respondents “Think back to the hypothetical scenario between you and your friend(s). Using a scale from 1 (not at all) to 7 (very much), please rate the extent to which you agree with the following statements.”

#### 2.2.3. Anticipated Sexual Stigma Scale (ASSS)

The ASSS, developed for the purposes of the current study, was created using methods similar to those employed in the development of the SWS. However, no validated scales were consulted; instead, members of the research team used feedback from relevant subreddits to generate a list of 13 items to be piloted for comprehensibility and grammar. All 13 items were retained, and the instructions and 7-point response scale replicated those used in the SWS. Sample items included “To what extent would you feel shame for engaging in the sexual activity?” and “To what extent would you expect friends/family to judge you for engaging in the sexual activity?” The scale demonstrated adequate internal consistency (*α* = 0.80).

#### 2.2.4. Demographics

This study utilized a demographics questionnaire that included questions regarding participants’ age, race, and threesome and sexual history. Additionally, we asked participants to report on their “sex at birth” using “male” and “female” as options.

### 2.3. Procedure

The current study was conducted in accordance with the principles outlined in the Declaration of Helsinki (World Medical Association, 2013). Ethical approval was obtained from Institutional Review Board at the University of Minnesota (STUDY00020589), ensuring that our study adhered to both national and international guidelines. After obtaining IRB approval, participants were recruited via 3Fun^®^ and Prolific^®^ to participate in a study exploring “willingness to engage in sexual activities.” All interested participants were required to read an informed consent form and provide electronic consent. Those consenting were then randomly assigned to read one of the eight vignettes and then completed the battery of questionnaires, which also included additional measures not relevant to the current study. The study took about 15 min to complete. Participants recruited from 3Fun^®^ were compensated with a one-month free VIP membership and participants recruited via Prolific^®^ were compensated with a $2.50 deposit into their account.

### 2.4. Data Screening and Cleaning

All data was screened and cleaned using standard procedures outlined by Tabachnick and Fidell (2019). In particular, no participants were missing more than 3.4% of their data, so listwise deletion was used to deal with missing values. Using z-score cut-offs of +/−3.00, no outliers were identified on any of the primary outcome variables (i.e., stigma, enthusiasm, reluctance). Additionally, skew was assessed by dividing the skew statistic by the skew standard error. None of the resulting skew z-scores demonstrated significant skew (+/−2.58).

## 3. Results

Descriptive analyses indicated that participants were fairly neutral when responding to both the Enthusiasm subscale (*M* = 4.53, *SD* = 2.04) and the Reluctance subscale (*M* = 4.42, *SD* = 1.65) of the SWS. Additionally, they reported anticipating moderate levels of stigma if accepting the sexual offer, as evidenced by a mean score of 3.60 (*SD* = 1.19) on the ASSS. Finally, comparative analyses revealed that the two samples did not differ on any of the primary variables in the study.

### 3.1. Anticipated Stigma

To partially examine H1, H2, and H3 with regard to anticipated stigma, an analysis of variance (ANOVA) was conducted with sex of the participant (male, female), type of behavior (dyadic, MST), and sexual makeup (same-, other-sex) as IVs and scores on the ASSS as a DV. See Table 1 for all means and standard deviations. The results indicated that all three main effects were significant. In particular, as expected (H1), female participants anticipated greater stigma (*M* = 4.28, *SD* = 1.12) than did male participants (*M* = 3.21, *SD* = 1.04), *F*(1, 531) = 99.34, *p* < 0.001, *η_p_*^2^ = 0.16. In contrast to H2, those receiving a dyadic sexual offer anticipated greater stigma (*M* = 3.76, *SD* = 1.27) as compared to those receiving an MST sexual offer (*M* = 3.51, *SD* = 1.14), *F*(1, 531) = 6.75, *p* = 0.01, *η_p_*^2^ = 0.01. Finally, consistent with H3, participants reported anticipating greater stigma about accepting a sexual offer that included a member of the same sex (*M* = 3.70, *SD* = 1.15 as compared to those that included all members of the other sex (*M* = 3.47, *SD* = 1.24), *F*(1, 531) = 10.57, *p* = 0.001, *η_p_*^2^ = 0.02.

However, these main effects were qualified by two two-way interactions. The first interaction was between sex of participant and sexual makeup, *F*(1, 531) = 5.09, *p* = 0.02, *η_p_*^2^ = 0.02. Results from a simple effects analysis indicated that the effect of sexual makeup was significant for male participants (*F*(1, 536) = 8.21, *p* = 0.004, *η_p_*^2^ = 0.02) but not female participants (*F*(1, 536) = 0.09, *p* = 0.76, *η_p_*^2^ = 0.00). In particular, males reported anticipating greater stigma about accepting a same-sex sexual offer (*M* = 3.35, *SD* = 1.07) as compared to an other-sex offer (*M* = 3.02, *SD* = 0.98).

The two-way interaction between type of behavior and sexual makeup was also significant, *F*(1, 531) = 7.40, *p* = 0.007, *η_p_*^2^ = 0.01. Again, a simple effects analysis was conducted and indicated that the effect of sexual makeup was significant for those considering a dyadic sexual offer (*F*(1, 536) = 18.73, *p* < 0.001, *η_p_*^2^ = 0.03) but not an MST offer (*F*(1, 536) = 0.08, *p* = 0.78, *η_p_*^2^ = 0.00). In essence, among those considering a dyadic sexual offer, greater anticipated stigma was reported when the offer involved a member of the same sex (*M* = 4.19, *SD* = 1.15) as compared to only a member of the other sex (*M* = 3.02, *SD* = 0.98).

### 3.2. Willingness to Accept the Sexual Offer

A separate ANOVA was conducted to investigate variations in willingness to accept the sexual offer using the current sample (H1, H2, and H3) using the same IVs. However, for this analysis, scores on the SWS were included as the DV (see Table 1). The results indicated that the main effect of sex of participant was significant (*F*(1, 524) = 120.43, *p* < 0.001, *η_p_*^2^ = 0.19). Consistent with H1, an examination of means suggested that male participants were significantly more willing to accept the sexual offer (*M* = 5.28, *SD* = 1.68) as compared to female participants (*M* = 3.24, *SD* = 1.98).

The effect of type of behavior was also significant, *F*(1, 524) = 11.86, *p* < 0.001, *η_p_*^2^ = 0.02). Contrary to H2, an examination of means suggested that participants were more willing to accept an MST sexual offer (*M* = 4.70, *SD* = 2.01) as compared to a dyadic sexual offer (*M* = 4.24, *SD* = 2.07). Finally, the main effect for sexual makeup was also significant, *F*(1, 524) = 60.67, *p* < 0.001, *η_p_*^2^ = 0.10. In line with H3, participants were more willing to accept a sexual offer that included only members of the other sex (*M* = 5.11, *SD* = 1.84) as compared to those that included a member of the same sex (*M* = 4.09, *SD* = 2.09).

These main effects were qualified by significant interactions. In particular, the two-way interaction between sex of participant and type of behavior was significant (*F*(1, 524) = 10.01, *p* = 0.002, *η_p_*^2^ = 0.02), with the results of a simple effects analysis revealing that the effect of type of behavior was significant for male (*F*(1, 536) = 9.47, *p* = 0.002, *η_p_*^2^ = 0.02) but not female participants (*F* (1, 536) = 0.87, *p* = 0.27, *η_p_*^2^ = 0.00). In essence, when reviewing the results for just the male participants, males were significantly more willing to accept the MST offer (*M* = 5.49, *SD* = 1.48) as compared to the dyadic sex offer (*M* = 4.86, *SD* = 1.96).

**Figure 1:**
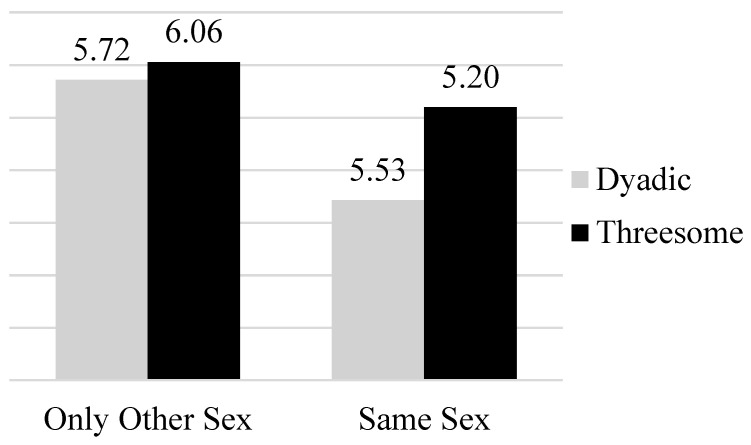
Bar graph depicting three-way interaction between type of behavior and sexual makeup for participants identifying as men.

However, all main effects and interactions were qualified by a significant three-way interaction between the sex of the participant, type of behavior, and sexual makeup, *F*(1, 524) = 7.05, *p* = 0.01, *η_p_*^2^ = 0.01. A follow-up simple effects analysis indicated that the interaction between type of behavior and sexual makeup was significant for males (*F*(1, 338) = 36.16, *p* < 0.001, *η_p_*^2^ = 0.05) but not females (*F*(1, 194) = 0.21, *p* = 0.65, *η_p_*^2^ = 0.00). When probing the interaction for male participants, it was clear that the type of behavior impacted reports of willingness to accept sexual offers that included a same-sex partner (*F* (1, 338) = 47.31, *p* < 0.001, *η_p_*^2^ = 0.12) but not for those that only included members of the other sex (*F*(1, 338) = 1.93, *p* = 0.17, *η_p_*^2^ = 0.01). See Figure 1 for a visual representation of the two-way interaction for male participants.

### 3.3. The Mediating Role of Anticipated Stigma

To explore the mediating role of anticipated stigma on willingness to accept the sexual offer (H4), three separate mediational models were conducted using the Hayes’ PROCESS macro for SPSS version 30 (Model 4). This approach was based on ordinary least-squares regression and the bootstrap method. In this study, 5000 bootstrap bias-corrected 95% confidence intervals (BC CIs) were used for mediation analyses and if a BC CI did not contain zero, the mediation was considered significant.

**Figure 2:**
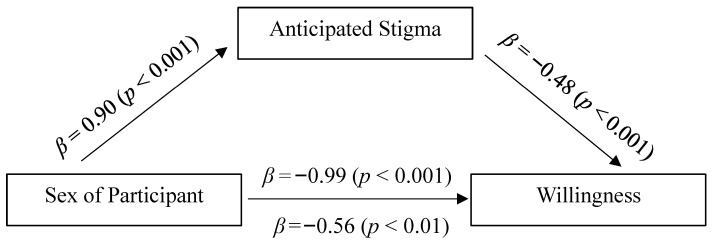
Mediational model for the effect of sex of participant.

In the first model, sex of participant was entered as the predictor (X), anticipated stigma as the mediator (M), and scores on the SWS as the outcome variable (Y). The results from this mediational analysis indicated that anticipated stigma partially mediated the effect of sex of participant on willingness (See Figure 2). After anticipated stigma was added to the model, the coefficient was reduced from *β* = −0.99 to *β* = −0.56, and the significance level decreased as well (from a *p* < 0.001 to a *p* = 0.01). The indirect effect was significantly different from zero; indirect effect = −0.43, SE = 0.05, 95% CI = [−0.52; −0.35]. The Sobel test provided further evidence that the reduction in the effect of sex of participant, after including anticipated stigma, was statistically significant (*Z* = −8.29, *p* < 0.001).

**Figure 3:**
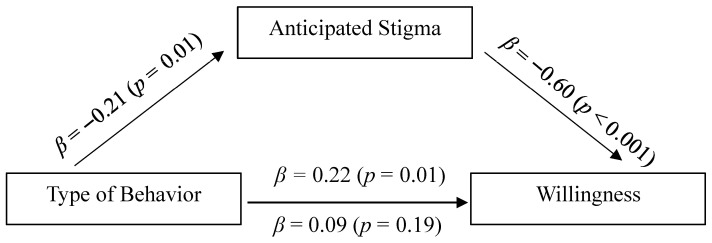
Mediational model for the effect of type of behavior.

A nearly identical approach was adopted in the second model; however, type of behavior was now included as the predictor variable (X). The results from this mediational analysis indicated that anticipated stigma fully mediated the effect of type of behavior on willingness (See Figure 3). After anticipated stigma was added to the model, the coefficient was reduced from *β* = 0.22 to *β* = 0.09, and the significance level decreased as well (from *p* = 0.01 to *p* = 0.19). The indirect effect was significantly different from zero; indirect effect = 0.13, SE = 0.05, 95% CI = [0.02; 0.23]. The Sobel test provided further evidence that the reduction in the effect of sex of participant, after including anticipated stigma, was statistically significant (*Z* = 2.32, *p* = 0.01).

**Figure 4:**
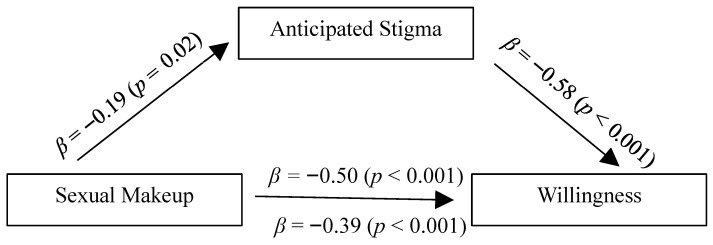
Mediational model for the effect of sexual makeup.

Finally, in our third model, sexual makeup was included as the predictor variable (X). The results indicated that anticipated stigma partially mediated the effect of sexual makeup on willingness (See Figure 4). After anticipated stigma was added to the model, the coefficient was reduced from *β* = −0.50 to *β* = −0.39. The indirect effect was also significantly different from zero; indirect effect = −0.11, SE = 0.05, 95% CI = [−0.21; −0.01]. The Sobel test provided further evidence that the reduction in the effect of sex of participant, after including anticipated stigma, was statistically significant (*Z* = −2.10, *p* = 0.02).

## 4. Discussion

The current study was designed to investigate gender differences in willingness to accept a variety of sexual offers (i.e., dyadic vs. multi-person sex) with same- and other-sex partners. Guided by Sexual Script Theory (Simon & Gagnon, 1984) and the backlash effect framework (Rudman, 1998), we hypothesized that women, as well as individuals invited to engage in more stigmatized sexual behaviors (e.g., MSTs, same-sex encounters), would demonstrate lower willingness as compared to men and those invited to engage in less stigmatized behaviors.

Overall, our results partially support the persistence of the traditional SDS and the gendered backlash effect (H1; Rudman, 1998). Male participants reported lower anticipated stigma and greater willingness to accept casual sexual offers than female participants. These results align with prior research suggesting that the SDS remains operative, particularly in evaluations of unconventional or nontraditional sexual behaviors (Gómez-Berrocal et al., 2022; González-Marugán et al., 2021; Thompson et al., 2018; Weber & Friese, 2024). They also extend prior work by Conley et al. (2013), indicating that women’s reluctance to engage in sexual behavior due to anticipated stigma is not limited to dyadic casual sex but may extend to encounters involving multiple partners and same-sex participation.

Although the main effect of behavior type was contrary to H2, both anticipated stigma and willingness to accept sexual offers varied as a function of behavior type and the sexual composition of the encounter. Examination of interaction effects revealed that participants anticipated the least stigma and expressed the highest willingness for dyadic encounters involving only other-sex partners. This pattern, however, was obscured in the main effects by the particularly high stigma and low willingness reported by men for same-sex dyadic encounters. These results are consistent with Sexual Script Theory and Rubin’s (1984) Charmed Circle framework, which conceptualizes sexual behaviors as existing within a hierarchy that privileges heteronormative, monogamous, procreative acts while marginalizing others. From this perspective, the observed effects of behavior type and sexual composition may represent a novel application of the backlash effect, in which individuals of all genders are less willing to engage in behaviors perceived as socially stigmatized.

Importantly, the current findings suggest that the backlash effect associated with participation in nontraditional sexual behavior disproportionately affected men’s anticipated stigma and willingness to accept same-sex offers, particularly in dyadic contexts with another man. This reluctance to engage in same-sex behavior among heterosexually identifying men is consistent with research documenting stronger bias against gay men compared to lesbian women (Chung & Katayama, 1996; Herek, 2004; Wright & Perry, 2006). In fact, a study conducted by Herek in 2004 indicates that gay men are more likely to be subject to negative stereotypes of weakness and femininity, whereas lesbian women are less often stereotyped as violating gender norms. Thus, the present findings support a novel application of the backlash effect, wherein heterosexual men’s reluctance to engage in same-sex behavior appears to be driven by the anticipation of sexual stigma.

The partial mediation effects observed for sex suggest that additional factors likely contribute to the relationship between participant gender and willingness. For example, women may perceive greater physical and reproductive risk in sexual encounters than men (e.g., risk of sexual assault or unintended pregnancy). Differences may also be shaped by the “pleasure gap,” wherein women report lower rates of sexual pleasure and orgasm compared to men (Armstrong et al., 2012). The mediation observed for sexual composition may also have been influenced by the vignette design. For some participants, the prospect of a sexual scenario involving a friend may have been more or less appealing depending on the nature of the relationship. Scoats’ (2020) qualitative work on threesomes suggests that both the composition of the group and whether partners are known or unknown can affect willingness, as motivations may differ between pursuing erotic novelty and engaging in a socially or emotionally significant sexual experience. Finally, it is likely that individual differences in sexual curiosity and openness to novel sexual experiences contributed to the mediation effects observed. Indeed, personality research indicates that individuals higher in Openness to Experience tend to report more frequent and varied sexual behaviors (McCrae, 1994).

### Limitations and Future Directions

The results of the study must be considered in light of some of the limitations. First, because participants were recruited via the app 3Fun^®^ and Prolific^®^, our sample was likely biased. In particular, adults recruited on these platforms are likely sexually permissive and erotophilic as compared to the population as a whole. To mitigate against recruitment bias in future studies, researchers should adopt more wide-reaching methods to collect data that better represents the broader population. Second, we used vignettes to describe a hypothetical scenario in which the participant was invited to engage in sexual behavior. Consequently, the use of these vignettes inherently limits the external validity of the results (Finger & Rand, 2003). Moving forward, future studies should be conducted to replicate our results using real-world scenarios. Third, although the vignettes were designed to approximate realistic scenarios, they inevitably simplified nuanced aspects of sexual decision-making. Notably, this study did not account for a range of potential moderators that may critically influence anticipated stigma and one’s willingness to engage in sexual behaviors. For example, perceived risk (e.g., STIs, pregnancy, physical safety), sensation-seeking tendencies, and socio-cultural attitudes toward MSTs were not systematically assessed. These variables may meaningfully moderate participants’ responses and should be examined in future research to better contextualize the findings. Finally, both the SWS and the ASSS were created for the purposes of the current study and underwent limited piloting for readability, grammar, and content clarity. Although both measures demonstrated strong internal consistency in the present sample, they have not been subjected to more rigorous validation procedures (e.g., confirmatory factor analysis, convergent and discriminant validity testing). In the future, researchers should seek to validate the SWS and ASSS across diverse populations, relationship contexts, and sexual orientations to ensure that observed effects in subsequent studies reflect those reported here.

## 5. Conclusions

Overall, the results from this study support the existence of the traditional SDS and a gendered backlash effect in regard to MST participation, as women anticipated more stigma and were less willing to accept offers for these behaviors than were men. However, this study also reveals a new gendered backlash effect whereby heterosexual men may be more reluctant than heterosexual women to engage in same-sex sexual behavior due to fears of being stigmatized. These findings expand our understanding of stigma and the SDS while introducing new nuance to our understanding of gendered backlash effects. Our findings have uncovered novel applications for the backlash effect, which we hope will draw attention to the power of stigma when explaining gender differences across sexual contexts. In particular, we hope that mental health professionals consider the findings of this research when working with clients who are struggling with fears of being stigmatized for engaging in nontraditional sexual behaviors, as the nuances of gendered backlash effects may provide useful insight into these struggles.

## Figures and Tables

**Table 1:** Means and standard deviations for all primary variables.

			Anticipated Stigma *M* (*SD*)	Willingness *M* (*SD*)
Male Participant	Other-Sex	Dyadic (*N* = 72)	3.00 (1.05)	5.44 (0.99)
		MST (*N* = 76)	3.03 (0.91)	5.73 (0.92)
	Same-Sex	Dyadic (*N* = 43)	3.95 (1.18)	3.71 (1.34)
		MST (*N* = 151)	3.18 (0.98)	5.06 (1.24)
Female Participant	Other-Sex	Dyadic (*N* = 41)	4.25 (1.22)	3.87 (1.23)
		MST (*N* = 45)	4.25 (1.28)	3.86 (1.65)
	Same-Sex	Dyadic (*N* = 37)	4.48 (1.05)	3.34 (1.43)
		MST (*N* = 75)	4.21 (1.01)	3.27 (1.31)

*Note.* Mean scores on the ASSS ranged from 1 to 7, with higher scores indicating greater anticipated stigma. Mean scores on the SWS also ranged from 1 to 7, with higher scores indicating greater willingness.

## Data Availability

Data is available on our OSF page (https://osf.io/95ebu/?view_only=c8030458590c4f60aa13f8420405ffcd, accessed on 10 June 2025).

## Supplementary Materials

The following supporting information can be downloaded at: https://www.mdpi.com/article/10.3390/bs15081128/s1.
