# Supplementary material for: Comparing Gender Differences in Willingness to Accept Same- and Other-Sex Dyadic and Multi-Person Sexual Offers: An Examination of the Backlash Effect
Source: Behav Sci (Basel). 2025 Aug 20;15(8):1128. doi: 10.3390/bs15081128 (PMC12383178; doi:10.3390/bs15081128)
Supplement: Supplementary file 1 [file behavsci-15-01128-s001.zip › behavsci-3720540-supplementary.pdf]

# Supplementary Materials

## Vignettes

*MST vignettes:*

MMF (Male participant)

Recently, you were hanging out with your friends and the topic of sexual experiences came up. They ask if you would be interested in having a threesome with them (a male and a female).

FFM (Male participant)

Recently, you were hanging out with your friends and the topic of sexual experiences came up. They ask if you would be interested in having a threesome with them (two females).

FFM (Female participant)

Recently you were hanging out with your friends and the topic of sexual experiences came up. They ask if you would be interested in having a threesome with them (a male and a female).

MMF (Female participant)

Recently you were hanging out with your friends and the topic of sexual experiences came up. They ask if you would be interested in having a threesome with them (two males).

*Dyadic vignettes:*

MM (Male participant)

Recently you were hanging out with your friend and the topic of sexual experiences came up. He asks if you would be interested in having sex with him (one male).

FF (Female participant)

Recently you were hanging out with your friend and the topic of sexual experiences came up. She asks if you would be interested in having sex with her (one female).

MF (Male Participant)

Recently you were hanging out with your friend and the topic of sexual experiences came up. She asks if you would be interested in having sex with her (one female).

MF (Female Participant)

Recently you were hanging out with your friend and the topic of sexual experiences came up. He asks if you would be interested in having sex with him (one male).
